# Correction: Highly photoluminescent nitrogen-rich carbon dots from melamine and citric acid for the selective detection of iron(iii) ion

**DOI:** 10.1039/d3ra90101g

**Published:** 2023-11-08

**Authors:** Shaoqing Liu, Ruili Liu, Xia Xing, Chongqing Yang, Yi Xu, Dongqing Wu

**Affiliations:** a Department of Chemical Engineering, School of Environment and Chemical Engineering, Shanghai University Shanghai 200444 China; b Department of Electronic Engineering, National Engineering Lab for TFT-LCD Materials and Technologies, Shanghai Jiao Tong University Shanghai 200240 China ruililiu@sjtu.edu.cn; c School of Chemistry and Chemical Engineering, Shanghai Jiao Tong University Shanghai 200240 China wudongqing@sjtu.edu.cn

## Abstract

Correction for ‘Highly photoluminescent nitrogen-rich carbon dots from melamine and citric acid for the selective detection of iron(iii) ion’ by Shaoqing Liu *et al.*, *RSC Adv.*, 2016, **6**, 31884–31888, https://doi.org/10.1039/C5RA26521E.

In the original manuscript, the authors regret an error in the vertical axis label for Fig. 3c. The vertical axis was mistakenly labelled as “Abs” rather than “F”. A correct Fig. 3 is provided below.

**Fig. 3:**
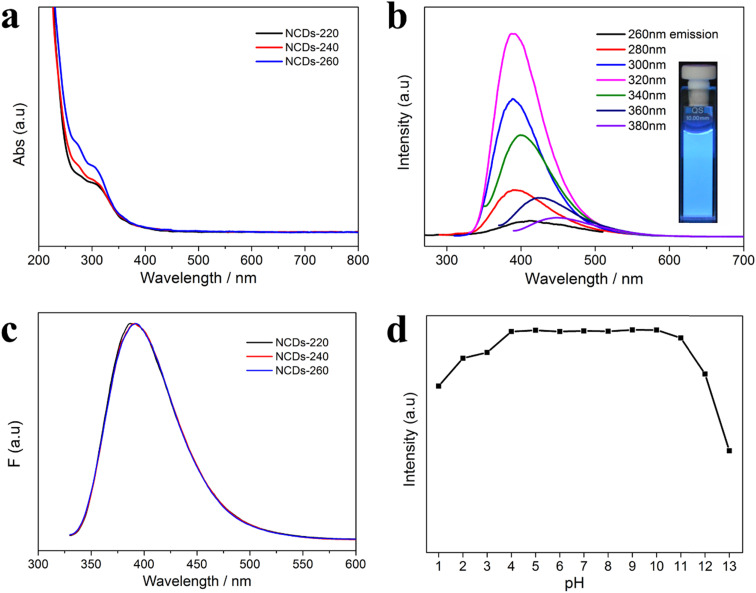
(a) UV/Vis absorption spectra of the N-rich CDs; (b) PL emission spectra of NCDs-240 with progressively increased excitation wavelengths from 260 to 380 nm with 20 nm increment. Inset of (b) is the optical image of NCDs-240 suspensions with strong blue luminescence under the excitation at 365 nm; (c) normalized emission spectra of N-rich CDs under the maximum excitation wavelength of 320 nm; (d) PL intensity of NCDs-240 in the solutions with different pH values.

The conclusions remain unaffected by this change.

The Royal Society of Chemistry apologises for these errors and any consequent inconvenience to authors and readers.

## Supplementary Material

